# Neurovascular Complications Following Clavicle Fracture Fixation: Timing, Mechanisms, and Clinical Implications

**DOI:** 10.7759/cureus.74512

**Published:** 2024-11-26

**Authors:** Aryan Borole, Diana Vitkovska, Jason Yang, John P Avendano, James Monica, Brian M Katt

**Affiliations:** 1 Department of Orthopedic Surgery, Rutgers Robert Wood Johnson Medical School, New Brunswick, USA

**Keywords:** arterial thoracic outlet syndrome, arteriovenous (av) fistula, clavicle fractures, neurological complications, subclavian pseudoaneurysm

## Abstract

This review addresses the neurovascular complications associated with the surgical treatment of clavicle fractures through open reduction and internal fixation (ORIF). Despite being a generally safe procedure, it can lead to severe complications including brachial plexopathy, pseudoaneurysm, arteriovenous fistulas (AVF), deep vein thrombosis (DVTs), and thoracic outlet syndrome (TOS). One significant observation, not often highlighted in previous literature, is that neurovascular complications are more common in cases involving delayed fixation, nonunion, or malunion, compared to those treated acutely. This review emphasizes the impact of the timing of surgery on the frequency of these rare complications and examines their underlying mechanisms.

## Introduction and background

Clavicle fractures account for 5%-10% of all adult fractures, primarily occurring in younger, active populations, with midshaft clavicle fractures comprising approximately 80% of all clavicle fractures [1]. While most midshaft clavicle fractures can successfully be treated non-operatively, surgical treatment should be considered in cases of symptomatic nonunion and in acute fractures with 100% displacement, 2 cm of shortening, significant comminution, or a Z-type pattern [1]. Complications arising from operative fixation are rare, but can be devastating, especially if not diagnosed quickly and managed appropriately.

Neurovascular complications are of particular concern with clavicle fixation due to the proximity of critical structures and can include brachial plexopathy, pseudoaneurysm, arteriovenous fistulas (AVF), deep vein thrombosis (DVTs), and thoracic outlet syndrome (TOS). An interesting observation not previously noted was that the frequency and etiology of these potential complications may also be affected by the time between injury and initial surgery, particularly in the case of nonunion or malunion where abundant callus may have compressive effects and the length of the clavicle may be altered. This finding is present in many cases of vascular complications but, according to the data, more pronounced in neurologic complications.

In this review, we provide a brief overview of the etiology, incidence, and mechanisms of various neurovascular complications following open reduction and internal fixation (ORIF) of clavicle fractures, with a particular focus on the time from injury to fixation and the timing of surgery relative to the development of symptoms.

## Review

Vascular complications

Iatrogenic vascular complications from clavicle fixation are incredibly rare. A meta-analysis of 582 cases did not report any vascular complications, concluding it would be very unlikely to establish a large enough study size to estimate a true incidence of these complications [1]. While uncommon, these complications tend to be clinically significant, potentially limb-threatening, and preventable. They may result in AVF, pseudoaneurysms, DVT, or TOS [2-5].

The documented causes of vascular injury during surgical procedures include tearing during mobilization of the bone fragments, air embolism resulting from vessel injury when removing the drill from the medial plate hole, venous air embolism associated with central venous catheterization, as documented in cases of hip arthroplasty, a prominent screw in the medial two-thirds of the clavicle, and over-reduction of the fracture in the presence of abundant callus and/or comminuted fragments [2,5-8].

*Arteriovenous* *Fistula*

AVFs are abnormal connections between arteries and veins. AVFs can be a result of a congenital anomaly or be secondary to iatrogenic injury or trauma caused by penetrating injury to the vasculature. There has been only one reported case of iatrogenic subclavian AVF as a complication of screw-and-plate osteosynthesis of clavicle fracture [2]. On the first postoperative day, a pulsatile mass and thrill were detected at the right supraclavicular region. The patient was discharged three days later and presented again three months later with right limb weakness and a growing mass. After a stent was placed and the plate removed, the postoperative course was unremarkable, and the patient made a full recovery.

AVFs are the result of penetrating injury to both an artery and vein causing the damaged artery and vein to meet each other at the site of injury. The damaged walls of the artery and vein begin to break down further, creating a tract allowing blood to flow directly from the high-pressure arterial system into the low-pressure venous system. Blood from the damaged artery now bypasses the normal capillary network and flows directly into the vein leading to alterations in blood flow dynamics and pressure within the affected vessels. An unaddressed AVF may lead to venous hypertension and subsequent congestive heart failure or limb-threatening ischemia. The authors of this case report recommend that the fracture reduction and hole drilling should be done with caution and the screw length must be measured carefully and accurately. While an extremely rare complication, it is crucial for surgeons to be aware of this uncommon occurrence [2].

Pseudoaneurysm

Pseudoaneurysms are defined as false aneurysms that occur at the site of arterial injury and, unlike a true aneurysm, are not contained by the arterial wall. Subclavian artery pseudoaneurysms are rare and usually occur due to an inadvertent arterial puncture during central venous catheterization. They should be suspected in posttraumatic injuries in a patient who presents with pulsatile mass in the clavicular region as well as peripheral embolization and brachial plexus compression.

In all the cases found related to operative fixation, the mechanism behind injury was the penetration of a medial screw into the subclavian artery. The length of the screw in the three cases where it was mentioned averaged 27.7 mm with a range of 26-30 mm, which is within the range of the reported clavicle diameter [9]. However, in the four cases that reported the length and direction of protrusion, the average length of protrusion was 8.7 mm posteroinferiorly with a range of 7-10 mm. This is concerning as previous studies have reported the mean distance between clavicle and vasculature as 17-26 mm. A 1996 study has proposed that the penetration occurs during arm movement when the vessels are brought closer to the screw tip [10].

Delayed subclavian pseudoaneurysms associated with clavicle ORIF have previously been described in the literature in eight cases (Table 1). In all reports, the patients presented with either limb claudication, swelling, and/or pain in the affected limb. Treatment modalities included endovascular repair, interpositional grafting, and open repair of the aneurysm [3,8,11-13]. From the eight cases of pseudoaneurysm, the average time of presentation of symptoms from the original surgery was just above 4.9 years, with a median time of about six years ranging from two weeks to 10 years [14,15]. It is possible that extensive collateral supply through the scapular anastomosis delays the presentation of symptoms. Across all articles, pseudoaneurysms tended to present more often after cases of delayed fixation or nonunion compared to acutely treated fractures. 

**Table 1:** Iatrogenic pseudoaneurysms as a result of clavicle ORIF following fracture ORIF: open reduction and internal fixation

Study	Cause	Acute ORIF vs delayed vs nonunion	Presentation	Screw length	Protrusion length/direction	Treatment	Outcome
Johnson (1996) [10]	Second most medial screw perforated artery	Acute	Claudication at 22 months	Not reported	8 mm posterior aspect of clavicle	Vascular graft	Return to full normal function. Moderate decrease in arterial pressure
Casselman (1997) [12]	Screw medial to fracture site perforates artery	Delayed ORIF	Claudication at 8 years	Not reported	Not reported	Vascular graft	Uncomplicated recovery
Shackford (2003) [3]	Screw lateral to the fracture site perforated artery	Nonunion	Paresthesias and aching pain at 8 years	30 mm	10 mm posteroinferiorly	Vascular graft	Uncomplicated recovery
Bain (2010) [8]	Medial screw perforated artery	Nonunion	Paraesthesia and claudication in his left hand at 6 years	26 mm	10 mm posteroinferiorly	Endovascular angioplasty balloon	Uncomplicated recovery
Pallett (2018) [13]	Protruding screw tip	Nonunion	Claudication 5 years post ORIF	Not reported	Not reported	Open repair of the aneurysm	Claudication symptoms resolved
Lewis (2019) [11]	2 medial screws perforated artery	Nonunion	Swelling and pain along the superior side of the clavicle, as well as numbness and tingling in the left arm in an ulnar nerve distribution at 2 weeks	27 mm	2 screws at the middle third of the clavicle extending 10 mm and 7 mm beyond the inferior cortex	Endovascular cannulation	Full recovery
Isse (2023) [14]	Protruding screw tip	Acute	Swelling in the superior side of the clavicle 2 weeks post-surgery	Not reported	Not reported	Ligature and subsequent bypass	Full recovery
Cogburn (2023) [15]	On X-ray, new lucency surrounding the middle screw and the most distal screw	Nonunion	A pulsatile right supraclavicular bulging mass without tenderness, 6 years after revision surgery and 4 years after ORIF	Not reported	7 mm anterior to the subclavian artery	Vascular stent	2 weeks after stenting patient developed sudden-onset paresthesias in the right hand. Found to have another brachial artery thrombosis

Venous Injury

Compared to pseudoaneurysms and arterial injuries, which are commonly diagnosed postoperatively, traumatic venous injuries are typically identified intra-operatively, except for DVT [16]. The first known venous complication took place in 1960 [6]. In a series of 18 cases of clavicle fixation in a single hospital, there was only one complication of this sort. In this case, the subclavian vein was torn during the freeing of the ends. The outcome was favorable, but little additional information was provided about the complication. No other cases of this complication have been reported since.

Another case of rare venous injuries is a venous air embolus that can prove fatal. In one case, while exposing the fracture site, the patient’s SpO_2_ suddenly dropped to 78% and the patient presented with sinus tachycardia, hypotension, hypocapnia, and a mill wheel murmur [17]. A similar episode occurred as the surgeon checked bleeding before closing the wound and the patient required less than 10 minutes to stabilize. In a second 2013 case, the patient developed a fatal air embolism as a result of vessel injury [7]. On withdrawal of the drill from the final, most medial screw hole, profuse low-pressure bleeding from a tear in the subclavian vein was noted. After the initial diagnosis of a pneumothorax was disproved, a central venous line was then inserted, and air was aspirated from the right atrium, suggestive of an air embolus. The coroner’s report marked the cause of death as "an air embolism and severe hemorrhage” because of “perforation of the right subclavian vein.” Venous air emboli remain a rare complication, but surgeons should maintain a high index of suspicion, given how quickly a patient can decompensate.

DVT is another rare complication of clavicle fixation. It was noted in one case, identified at a three-week follow-up after the patient complained that his arm had enlarged over the previous week and had become painful and heavy [18]. The upper limb DVT symptoms resolved after three months of anticoagulation treatment. The cause of the DVT was not known but it was speculated to be either caused by the surgery or an underlying TOS by venous thrombosis such as Paget-Schroetter syndrome [9].

Thoracic Outlet Syndrome

TOS results from compression of the brachial plexus, the subclavian artery, and the subclavian vein in the thoracic outlet region [19]. Classically, TOS occurs in the scalene triangle, but in the setting of clavicle fixation, the costoclavicular space is of greater concern.

Causes for TOS after clavicle fixation can include: impingement of hardware on the thoracic outlet (TO), callus formation compressing onto the TO, or an excessively long screw [16,19-21]. With the exception of one case where the patient presented with TOS five months after their operation, TOS tends to present nearly immediately after surgery [21]. In the one case where TOS presented after five months, it was hypothesized that it might not have a true iatrogenic cause as this time symptoms tend to be more typical of TOS following non-operative management. The compression may have been the result of callus forming after fixation as opposed to the mobilization of a pre-existing callus after the reduction [4].

In a 1997 report, the authors remark that many complications occurred after osteosynthesis with Kirchner wires, which can migrate and penetrate other areas [12]. Their case report was the first to report an incident of TOS because of plate osteosynthesis and stated that their patient’s symptoms were due to a protruding screw. While this was the first mention of this cause of TOS, there have been other reports in more recent years. In a 2024 case report, a team of vascular surgeons wrote about the possibility of chronic vessel damage with resultant thrombo-embolism because of incongruent clavicle screw lengths resulting in impingement of the arterial adventitia and primary arterial TOS [16]. The incorrectly sized screw resulted in left mid-subclavian artery thrombosis with limb-threatening distal embolization to the brachial artery and forearm vessels seven years after surgery. While this is one of the more common causes of TOS, the initial prognosis tends to be favorable following removal of the impinging object such as the plate/screw construct as well as a potential course of anticoagulation therapy [16,19].

Brachial plexus and neurological injuries

Anatomy and Overview

Due to the proximity of the brachial plexus to the posterior clavicle, operative management of clavicular fractures presents a risk of injury to the brachial plexus from stretching during reduction or clavicular lengthening, compression by fracture fragments, prominent callus, implants, or vascular pathology [22]. Although the mechanisms of injury to the brachial plexus can be variable, they can be generally categorized into injuries caused by traction or compression.

Injuries to the brachial plexus due to ORIF of a fractured clavicle are rare, with incidence rates ranging from 0 to 1.5% [9]. However, if not addressed promptly, restoring proper function is challenging and can lead to severe deficits in upper limb function [23].

Traction Injuries

The formation of scar tissue during the fracture healing process can lead to adherence of surrounding tissues including the brachial plexus to the fracture site. Due to the presence of the fibrotic attachments between the neurovasculature and the bony fragments, their maneuvering during intra-operative reduction can lead to traction force imposed on the brachial plexus. Table 2 below outlines the current literature on traction injuries of the brachial plexus. 

**Table 2:** Iatrogenic brachial plexus traction injuries following clavicle ORIF ORIF: open reduction and internal fixation

Study	Original diagnosis	Acute vs nonunion vs delayed ORIF	Complication	Cause	Presentation	Treatment	Outcomes
Ring (2005) [24]	Acute mid-clavicle fracture	1 Acute and 2 Delayed	1 upper trunk and 2 complete brachial palsies	Delivery of the fracture ends from the wound to facilitate reaming	Weakness and pain	Observation	Full recovery within 6 months
Jeyaseelan (2013) [25]	Displaced or minimally displaced mid-clavicle, and medial and lateral fractures	Delayed	Plexopathy in all, 4 cases of ruptures of C5 and C6	Traction of the nerve bundle adherent to the posterior surface of the fracture site	Pain, weakness, and reduced sensation	All cases: neurolysis. 2 nerve transfer, and 2 nerve grafting	Pain resolution at 12 months
Gross (2013) [26]	Displaced mid-diaphyseal clavicle fracture with shortening and comminution	Delayed	Acute brachial plexopathy of middle trunk predominantly posterior cord	Traction injury upon re-establishment of the clavicular length	Hand weakness and difficulty with upper extremity movement	Hardware removal and re-establishment of clavicular length reduction	Satisfactory recovery at 8 months
Johnson (2020) [27]	Allman Type I comminuted fracture of clavicle	Delayed nonunion	Partial motor and sensory traction neuropraxia	Clavicular lengthening after fracture fixation	Paresthesia, numbness, and weakness	Close monitoring and occupational/physical therapy	Full neurologic recovery with mild residual neuralgias in one year

In a series of 21 cases, brachial plexus injury occurred following delayed plate fixation of the clavicle fractures with the mean time from injury to fixation of 19 days. The etiology of the plexopathy in 20 cases was explained by traction of the nerve bundle adherent to the posterior surface/undersurface of the fracture site. A higher degree of comminution was found to be related to increased adherence to the fibrotic scar [25]. In the remaining case, the injury was caused by traction due to direct tethering between the plexus and two screws at the fracture site [25]. In all cases, patients were treated with neurolysis. Ruptures of C5 and C6 were found in four patients and treated with spinal accessory nerve transfer in two cases. In the other two cases, medial cutaneous nerve grafting was used. At 12 months follow-up, pain resolved in all patients. In cases of delayed fixation, Jeyaseelan et al. recommend that before fracture fixation, thorough dissection of adherent soft tissues from the undersurface clavicle must be performed [25].

In the case of a displaced mid-diaphyseal clavicular fracture with significant shortening and comminution, restoration of appropriate clavicular length resulted in the development of traction neuropathy [26]. The surgery was performed at five weeks post-injury followed by acute onset of hand weakness and difficulty with movement in the affected arm 24 hours after surgery. Retrospective clavicular length comparison showed 27 mm lengthening immediately post ORIF and 29 mm shortening after hardware removal. Electromyography (EMG) performed one month after hardware removal demonstrated signs of axonotmesis, most significantly affecting the posterior cord. The exact etiology of the complication is not clear, but Gross et al. propose that during the five-week delay following the fracture before surgical intervention, the brachial plexus cords contracted in response to reduced infraclavicular space [26].

Another etiology of traction injuries to the brachial plexus results from the clavicular lengthening after fracture fixation. Johnson et al. report a case of brachial plexopathy following anterior-inferior plating and iliac crest bone graft for symptomatic atrophic nonunion at five months post-initial injury [27]. At 48 hours postoperatively, the patient experienced worsening numbness in an ulnar distribution and progressive weakness of the affected arm. A comparison of pre-and post-operative imaging revealed a 5.9 mm lengthening of the clavicle during ORIF, suggesting a traction neuropraxia may have occurred with intra-operative lengthening of the clavicle. At six months, the patient’s strength and sensation returned to pre-operative levels [27].

Ring and Holovacs reported transient brachial plexus palsies presenting acutely after two cases of delayed and one case of acute intramedullary fixation of the clavicle [24]. In two cases, upper trunk palsy was apparent after surgery with adequate alignment of the fracture and the implant on the radiographic imaging. The third patient had complete impairment of the brachial plexus with absent sensory and motor function. In all cases, within six months the patient recovered fully without intervention. The authors believe that injury to the brachial plexus likely occurred with the delivery of the fracture ends from the wound to facilitate reaming [24].

In short, traction injuries of the brachial plexus are reported following clavicle fracture fixation, particularly in delayed cases. Common mechanisms include nerve adherence to fibrotic scar tissue, traction during fracture reduction, and clavicular lengthening. Effective management often involves neurolysis, with nerve transfers or grafting in severe cases. Key findings highlight that delayed fixation and high fracture comminution increase the risk, and thorough soft tissue dissection is crucial to prevent these injuries.

Compression

Injury to the brachial plexus can occur in the setting of impingement by malunited bone fragments or compression by a prominent callus. Clavicular shortening and narrowing of the costoclavicular space as a result of the fracture fixation have also been reported to lead to the compression of the brachial plexus between the clavicle and the first rib as shown in Table 3. 

**Table 3:** Iatrogenic brachial plexus compression injuries following clavicle ORIF TOS: thoracic outlet syndrome; MRC: Medical Research Council; ORIF: open reduction and internal fixation

Study	Original diagnosis	Delayed ORIF vs nonunion	Complication	Cause of compression	Presentation	Treatment	Outcome
Namdari et al. (2012) [22]	Displaced midshaft clavicular fracture nonunion	Nonunion following delayed ORIF	Diffuse brachial plexopathy of predominantly lower trunk	Bone fragment	Decreased sensation, pain, and motor deficits	Bone fragment excision	Immediate pain resolution and improved sensation, motor function improvement at 4 months
Jeyaseelan et al. (2013) [25]	Displaced or minimally displaced mid-clavicle, medial and lateral fractures	Delayed ORIF	TOS	Clavicular shortening	Pain, weakness, and reduced sensation	All cases: neurolysis. 2 nerve transfer, 2 nerve grafting	Pain resolution at 12 months
Thavarajah and Scadden (2013) [28]	Minimally displaced midshaft clavicle fracture	Nonunion	Iatrogenic traction neurapraxia	Hypertrophic callus	MRC grade 0/5 power from C5–T1 and absent two-point discrimination	Callus and middle third of the clavicle removal and neurolysis of the plexus	MRC grade 4/5 power from C5–T1 and a return of two-point discrimination
Rosati et al. (2013) [29]	Displaced middle third clavicle fracture	Nonunion	Iatrogenic compartment syndrome	reduction of costoclavicular space	Paralysis of the median nerve, radial and ulnar nerves, paresthesias in C5-C6-C7-C8 roots distribution	Implant removal	Complete recovery at 3 months
McGilliray et al. (2022) [23]	Clavicular nonunion fracture	Nonunion	Compression brachial plexopathy	Between callus and first rib	Numbness and weakness	Hardware removal, brachial plexus decompression, and callus removal	Near complete recovery with residual muscle weakness

One case showed compression of the brachial plexus due to a malunited bone fragment initially missed on postoperative X-ray, with symptom resolution upon removal of the fragment [19]. The patient presented seven months post-injury with a displaced midshaft clavicular fracture nonunion. Four days after an ORIF and iliac crest bone graft was performed, CT showed a 2 cm malunited posteroinferior fragment compressing the brachial plexus not previously evident on radiographs and MRI. During fragment excision, the bone fragment was visualized above nerve tissue. The hardware was left intact. 

McGilliray et al. presented two case reports of brachial plexopathy following clavicular ORIF for nonunion due to prominent callus formation along the deep posterior surface of the clavicle [23]. In the first case, a patient presented with acute onset numbness and weakness in the affected arm in less than 12 hours post-op while in the second case, the patient presented with brachial plexopathy with one one-year history of clavicular nonunion originally treated with ORIF. In both cases, the patients underwent successful callus excision at the posterior and deep surface of the clavicle to relieve plexus compression against the first rib. Patient improvement following decompression surgeries suggests that urgent surgical intervention may be advantageous in the treatment of brachial plexopathies following clavicle ORIF [23].

One unique case reported iatrogenic postoperative brachial plexus compression from hypertrophic callus [28]. Following four months of conservative treatment for a minimally displaced midshaft fracture without neurovascular impairment, radiographic imaging revealed a significant callus and a hypertrophic nonunion. Eight months after the injury the patient underwent ORIF with autograft from the hypertrophic callus site. On post-surgical evaluation, the patient showed signs of incomplete brachial plexus injury with motor and sensory paresis. On surgical exploration, significant callus compression was found. The callus was removed along with the middle third of the clavicle, followed by neurolysis of the plexus [28].

Rosati et al. reported a unique case of what they describe as “iatrogenic compartment syndrome” [29]. A patient who was treated for a displaced middle third clavicle fracture of four months duration, presented with signs of brachial plexus injury immediately after surgery. The patient was treated conservatively, but after one month, post-surgery complete brachial plexus denervation was shown on EMG/NCV. Unlike in the previously discussed cases, in this patient, no callus formation was found to have been potentially causing compression. The authors explain the etiology of this patient’s plexopathy by surgical recreation of the Wright test that resulted from the reduction of the costoclavicular space by the rigid fixation of the fracture. During the Wright test retroposition and abduction of the shoulder reduces the costoclavicular space by 50%. In this case, following the initial fracture shortening of the clavicle led to the relaxation of the surrounding soft tissue including the brachial plexus. During the reduction, the re-establishment of the original length caused a lateral stretch of the costoclavicular space.

Previously discussed cases reported by Jeyaseelan et al. also presented similar etiology of post-surgical TOS in five patients. Upon clavicular fixation over 0.5 cm of shortening was recorded leading to a significant narrowing of the costoclavicular space causing compression of the brachial plexus between the clavicle and the first rib [25].

In summary, brachial plexus compression can result from malunited bone fragments, prominent callus formation, or costoclavicular space narrowing following clavicle fracture fixation. Cases highlight diverse etiologies, including bone fragment impingement and hypertrophic callus. Treatment strategies like fragment or callus removal and brachial plexus decompression typically yield significant recovery. Key findings emphasize that delayed fixation and excessive clavicular shortening increase compression risks. Prompt recognition and intervention are critical for optimal outcomes.

## Conclusions

Despite the high frequency of clavicle fractures, there is still debate about the proper choice of treatment. The overall rate of all complications following ORIF of acute displaced midshaft fractures was found to be 27.3%, with 9.1% of patients requiring a reoperation, though the rate of neurovascular complications is much less than this figure. An analysis comparing the non-operative and operative treatment of midshaft clavicle fractures found the nonunion rate to be 14.5% and 1.4% respectively. Surgical treatment has become commonplace following the results of the 2007 COTS study, which showed that the surgically treated group with superior plates had lower rates of nonunion and malunion, as well as a faster time to union, compared to conservative treatment. Although many clavicular fractures heal with some degree of deformity, most patients with malunion recover well barring a visible deformity. In these rare occasions, an exuberant callus can produce a local compression on the underlying brachial plexus, which may resolve as the callus matures. When symptomatic, malunion can result in loss of strength, fatigue, cosmetic complaints, and numbness. No studies detailing the exact rate of neurovascular complications from malunion following conservative treatment were found, and more work quantifying complication rates would better allow us to compare the safety of conservative and operative treatment with respect to this rare subtype of injury.

One limitation of this review is the usage of case reports and small series, which may not provide a comprehensive understanding of the true incidence and risk factors for neurovascular complications following clavicle fracture fixation. Given the rarity of these complications, large-scale studies or multicenter databases would be difficult to attain, yet necessary to better quantify their prevalence and identify consistent risk factors. Additionally, this review primarily focuses on postoperative complications without discussing long-term follow-up or patient outcomes in greater detail. Further research is needed to explore the long-term effects of these complications, the efficacy of various treatment options, and the role of pre-operative planning in minimizing risks.

Based on the findings of this review, surgeons should consider several strategies to mitigate neurovascular complications associated with clavicle ORIF. Pre-operative planning is critical, particularly in patients with delayed presentations or nonunion, as significant callus formation may necessitate more extensive dissection, increasing the risk of neurovascular injury. For patients with chronic shortening of the clavicle, minimizing excessive lengthening during fixation is recommended to reduce traction on the brachial plexus. Intra-operatively, surgeons should prioritize careful soft tissue handling and meticulous dissection to avoid traction injuries caused by adherence of the brachial plexus to fracture sites, especially in cases with significant fibrosis or delayed fixation. Accurate measurement of screw lengths is essential to ensure they do not protrude posteroinferiorly into neurovascular structures, and intra-operative imaging should be used to confirm proper hardware placement. Additionally, surgeons should avoid over-reduction or over-compression of fractures, which can compromise the costoclavicular space and increase the risk of TOS. Postoperatively, detailed evaluations are crucial, particularly in high-risk cases, to identify early signs of neurovascular compromise such as pseudoaneurysm, AVF, or brachial plexopathy. When neurovascular injury is suspected, imaging modalities like duplex ultrasonography or CT angiography should be employed, even if patients are initially asymptomatic. These recommendations aim to enhance patient safety and optimize outcomes by addressing the rare but severe complications identified in the review.
